# Characterization of HIV-1 Infection and Innate Sensing in Different Types of Primary Human Monocyte-Derived Macrophages

**DOI:** 10.1155/2013/208412

**Published:** 2013-01-28

**Authors:** Elisabeth A. Diget, Kaja Zuwala, Randi K. Berg, Rune R. Laursen, Stine Søby, Lars Østergaard, Jesper Melchjorsen, Trine H. Mogensen

**Affiliations:** ^1^Department of Infectious Diseases, Aarhus University Hospital, Skejby, 8200 Aarhus, Denmark; ^2^Department of Clinical Medicine, Aarhus University, Skejby, 8200 Aarhus, Denmark

## Abstract

Macrophages play an important role in human immunodeficiency virus (HIV) pathogenesis and contribute to establishment of a viral reservoir responsible for continuous virus production and virus transmission to T cells. In this study, we investigated the differences between various monocyte-derived macrophages (MDMs) generated through different differentiation protocols and evaluated different cellular, immunological, and virological properties. We found that elevated and persistent HIV-1 pWT/BaL replication could be obtained only in MDMs grown in RPMI containing macrophage colony-stimulating factor (M-CSF). Interestingly, this MDM type was also most responsive to toll-like receptor stimulation. By contrast, all MDM types were activated to a comparable extent by intracellular DNA, and the macrophage serum-free medium-(Mac-SFM-)differentiated MDMs responded strongly to membrane fusion through expression of CXCL10. Finally, we found that HIV infection of RPMI/M-CSF-differentiated MDMs induced low-grade expression of two interferon-stimulated genes in some donors. In conclusion, our study demonstrates that the differentiation protocol used greatly influences the ability of MDMs to activate innate immune reactions and support HIV-1 replication. Paradoxically, the data show that the MDMs with the strongest innate immune response were also the most permissive for HIV-1 replication.

## 1. Introduction

Infection with human immunodeficiency virus type-1 (HIV-1) is a worldwide pandemic with more than 33 million people estimated to be infected worldwide [1]. HIV is a human retrovirus that targets cells within the immune system and establishes lifelong infection resulting in immunodeficiency and the development of acquired immunodeficiency syndrome (AIDS) in untreated individuals [2]. The two main cellular targets of HIV-1 are CD4+ T cells and macrophages, although these two cell types display different characteristics of infection, including differences in viral uptake, the rate of HIV replication, cell fate, and capacity to form viral reservoirs [3]. Macrophages infected with HIV-1 represent a stable viral reservoir with continuous virus production, thus contributing to the spread of HIV-1 to other cells and to the immune pathogenesis [4].

Macrophages belong to the innate immune system, and their important role in the first line of defense against invading pathogens has been recognized since long [5]. Together with other innate immune cells, such as dendritic cells (DCs), macrophages are involved in early control of infections at portals of entry for microbes (i.e., skin, genital and gastrointestinal mucosa, and airways), where microbes are sensed via pattern recognition receptors (PRRs). This restricts viral replication via early upregulation of antiviral mediators, such as interferons (IFNs) and IFN-stimulated genes (ISGs). Stimulation of innate immune responses in macrophages via PRRs is generally well established to confer viral restriction in this cell type [6]. However, HIV does not trigger significant immune activation in macrophages, hence preventing sufficient cell-intrinsic inhibition of viral replication [7]. The lack of innate immune stimulation in the course of HIV infection is caused by a number of innate immune evasion strategies employed by HIV-1, including protease-mediated sequestration of RIG-I and Vpu-dependent depletion of interferon regulatory factor 3 [8–11]. The central role played by macrophages and the innate immune system during HIV-1 infection emphasises the importance of understanding the interaction between HIV-1 and macrophages.

Macrophages are a highly heterogenic cell population with cellular properties strongly influenced by the factors present during differentiation of the macrophage from the precursor cell, the monocyte. Indeed, monocytes are refractory to HIV infection and only become susceptible to infection after differentiation into macrophages [12]. It has been previously reported that the cytokines macrophage colony-stimulating factor (M-CSF) and granulocyte macrophage colony-stimulating factor (GM-CSF) differentially impact on the functions of macrophages if present during the monocyte-to-macrophage differentiation [13]. M-CSF-differentiated macrophages have a rather anti-inflammatory cytokine profile and have been used as a model for tissue macrophages, whereas GM-CSF-differentiated macrophages are directed into a more proinflammatory cytokine profile and may be used as a model system for DC development and function [14–16]. Thus, both cell type specific factors, like transcription factors and micro RNAs, as well as environmental conditions, such as cytokine production, immune responses, cellular activation, and differentiation, affect the ability of macrophages to support HIV replication [3].

HIV targets CD4+ T cells and macrophages, which become infected after a viral entry process involving interaction between the viral envelope gp120 protein and a cellular CD4-coreceptor complex [3, 4]. The co-receptor is either CXCR4 or CCR5, and the co-receptor usage influences the cell types infected. Although CCR5 and CXCR4 are both present on macrophages, this cell type is most frequently infected by R5 strains, possibly because R5 strains can exploit low levels of CD4 and/or CCR5 to enter macrophages [17]. After interaction between HIV gp120 and cellular CD4 and CCR5/CXCR4, viral internalization takes place through receptor-mediated endocytosis and viral access to the target cell cytoplasm. In addition, cell surface lectins, like mannose receptor and DC-SIGN, also bind to HIV gp120 and facilitate virus attachment, binding, or fusion in macrophages during endocytic viral uptake [4, 18]. However, HIV-1 can also enter macrophages through a receptor-independent alternative macropinocytosis pathway [10, 11], which is affected by cell activation status [12]. Receptor-independent endocytic virus uptake is dominated by nonproductive infection and limited innate immune signaling [19]. Endocytosed virions are mostly degraded during transit in the endocytic compartment, although, in certain cases, fusion after endocytosis and productive infection may ensue [20].

Innate immune activation through PRRs is believed to play a role in the immune response to HIV infection, and TLRs are well established to evoke inflammatory responses during infections with opportunistic pathogens during HIV-1 infection [21]. Moreover, it has been reported that HIV-1 can stimulate macrophage activities independent of TLRs [22], and in certain cell types, cytosolic PRRs, including RIG-like receptors (RLRs) and DNA sensors, may be operative [2, 10, 23]. During HIV infection, PRR activation can be mediated by pathogen-associated molecular patterns (PAMPs) directly derived from HIV, such as genomic HIV RNA recognized by endosomal TLR7/8 [24] or cytosolic RLRs [2, 10]. In addition, reverse transcription products, such as single-stranded DNA, may be recognized by cytosolic DNA sensors [23, 25]. Alternatively, PAMPs may be derived from opportunistic infections or originate from bacterial translocation across gastrointestinal mucosa, which has been demonstrated for LPS and CpG DNA, activating TLR4 and TLR9, respectively [6, 26–28]. The consequences of PRR activation during HIV infection are at least twofold. First, activation of proinflammatory antiviral pathways may restrict HIV replication through the induction of IFN and ISGs, particularly restriction factors including APOBEC3G, tetherin, and viperin [1, 5, 6]. Second, PRR stimulation may result in activation of proinflammatory responses mediated through nuclear factor-*κ*B (NF-*κ*B), which has the capacity to drive/activate HIV transcription through binding to the HIV long terminal repeat [29, 30]. For example, IFN-inducing molecules or pathogens that trigger TLR3, TLR4, and TLR7/8 have been demonstrated to impair viral replication [6, 31], whereas pathogens triggering TLR2 may increase virus production [32, 33]. Therefore, although HIV itself appears to display a striking absent, or at least only very limited, induction of IFN, ISGs, and NF-*κ*B during infection, stimulation of the innate immune response by non-HIV PAMPs through various PRRs may have important and profound influences on viral replication and establishment of infection.

In the present work, we have used human primary monocyte-derived macrophages (MDMs) differentiated through four different sets of conditions to evaluate the effect of the differentiation protocol on HIV-1 replication, PRR responsiveness, and HIV-1-induced ISG expression. The results demonstrate that the differentiation procedure strongly impacts on the ability of the MDMs to support HIV-1 replication, respond to PRRs, and to induce ISGs during HIV-1 infection. Our results emphasize that the choice of macrophage model system is important for evaluation of innate immune responses in general, including virus-cell interactions during HIV-1 infection.

## 2. Materials and Methods

### 2.1. Isolation and Culture of Monocyte-Derived Macrophages

 The buffy coats used for PBMC isolation were derived from healthy volunteer blood donors in Denmark with an ethical permission obtained from the Regional Ethics Committee (Project 108). Human monocytes were isolated using Ficoll-Paque purchased from GE Healthcare, Hillerød, Denmark. Briefly, the blood was diluted 1 : 1 with phosphate-buffered saline (PBS) (Lonza, Basel, Switzerland), placed under a layer of Ficoll-Paque and centrifuged for 15 min at 600 ×g. Cells from the interphase layer were harvested, washed twice with PBS, and resuspended in RPMI1640 with 50 U/mL penicillin and 50 U/mL streptomycin (Invitrogen, Glostrup, Denmark). The cells were then seeded in 6-well plates (Nunc, Roskilde, Denmark), 10 × 10^6^ cells per well. After 1 hour, the cells were washed 3 times with warm PBS before adding 1.5 mL differentiation medium to each well. The differentiation medium was either macrophage serum-free medium (Mac-SFM) with 50 U/mL penicillin, 50 U/mL streptomycin and either 10 ng/mL granulocyte macrophage colony-stimulatory factor (GM-CSF) or 20 ng/mL macrophage colony-stimulatory factor (M-CSF) (all Invitrogen, Glostrup, Denmark), or RPMI (Lonza, Basel, Switzerland) with 2 mM L-glutamine, 50 U/mL penicillin, 50 U/mL streptomycin, 10% human AB serum (Invitrogen, Glostrup, Denmark), and either 10 ng/mL GM-CSF or 20 ng/mL M-CSF. Cells were kept for 7 days in either medium to differentiate MDMs. Donor A-D in Figure 2 was also used as four of six donors included in the dataset presented in Figures 3 and 4. 

### 2.2. Virus Production

The R5 strain HIV-1 pWT/BaL (NIH AIDS Research and Reference Reagent Program, Bethesda, USA) was produced using a calcium chloride method in 293 T cells, in which we combined 10 *μ*g DNA plasmid in 450 *μ*L sterile water with 50 *μ*L 2,5 M CaCL_2_ and then added 500 *μ*L Hepes. The following day, the medium was changed, and 48 h and 72 h after transfection, the medium was harvested and filtrated through a 0.22 *μ*m filter. TCID50 of the virus was determined by using titration of the virus on TZM-bl cells (obtained from NIH AIDS Reagent Program, catalogue no. 8129) with Britelite Plus Reagent (PerkinElmer Inc.) for quantification of the luminescent signal. The calculations of TCID50 were then done using Reed Munch calculation. In parallel with HIV-1 pWT/BaL production, a control plasmid pUC19 was also transfected into 293 T cells and subjected to the same procedures as the HIV-1 strain. No virus particles were produced from the pUC19 plasmid, but the samples were used as a control for potential contaminant derived from the 293 T cells, from the pUC19 plasmid or plasmid preparations. Sendai virus (strain Cantell, kindly provided by Ilkka Julkunen, Helsinki) was grown in 11-day-old embryonated hen eggs, as previously described [34]. The infectivity titer of the virus in DCs was 4 × 10^9^ PFU/mL [35]. UV inactivation of the virus was performed by exposing the virus to UV light for 20 min unless otherwise indicated. The uninfected hen egg allantoic fluid did not stimulate proinflammatory cytokine expression in DCs, and the virus preparation did not contain lipopolysaccharide (data not shown).

### 2.3. MDM Stimulation with HIV-1 pWT/BaL

Briefly, after 7 days of differentiation, the medium was changed to a medium that did not contain GM-CSF or M-CSF. The concentrated virus was mixed with the appropriate media and added to the wells to a concentration of 5,000 × TCID50. The same amount from the pUC19 stock was mixed with the correct medium as a control. Medium with neither HIV-1 pWT/BaL nor pUC19 was added as an untreated control. The plates were placed in an incubator at 37°C with 5% CO_2_ for 4 hours, after which the medium was removed and each well was washed 3 times with approximately 1 mL PBS. Following this, 1.5 mL of the appropriate medium was added to the cells in each well. A washing control was included, in which MDMs were treated exactly like the rest, with the exception that the medium was removed immediately after addition. After washing, the plates were again placed in an incubator at 37°C with 5% CO_2_. In the following days, the supernatants were harvested on days 2, 4, 7, 10, and 13 after stimulation with virus. Evaluation of HIV-1 levels in the supernatants were made using HIV-1 p24 ELISA, as described previously [36].

### 2.4. MDM Stimulation by Pathogen-Associated Molecular Patterns (PAMPs)

The cells were differentiated for 7 days before the medium was changed to one without GM-CSF or M-CSF. Part of the cells was stimulated with LPS 100 ng/mL (InVivoGen, Toulouse, France), R848 (5 *μ*g/ml, InVivoGen, Toulouse, France), or cell media alone. The remaining cells were transfected with poly(dA : dT) 1.7 *μ*g/mL (Sigma Aldrich or InVivoGen) using lipofectamine2000 added in a ratio of 1 : 2.5 after manufacturer's protocol (Invitrogen, Glostrup, Denmark). A mixture using the same amount of lipofectamine2000 and Opti-MEM without poly(dA : dT) was used as control. After stimulation, the plate was placed in an incubator at 37°C with 5% CO_2_ for 6 hours before the harvest of the supernatants, which were then frozen to be used for cytokine analysis.

### 2.5. Detection of ISG Synthesis in MDMs

The MDMs were differentiated as described previously in RPMI M-CSF or Mac-SFM GM-CSF for 7 days before the medium was changed to one without the respective growth factors. The MDMs were stimulated with HIV-1 pWT/BaL. The concentrated virus was mixed with the appropriate media and added to the wells to a final concentration of 5000 × TCID50. 100 U/mL of IFN*α* (PBL, InterferonSource New Jersey, USA) and SeV MOI 0.5 were used as controls. Medium with neither HIV-1 pWT/BaL was included as untreated control. The plates were placed in an incubator at 37°C with 5% CO_2_ for 6 hours before they were harvested using trizol (Invitrogen, Glostrup, Denmark).

### 2.6. CXCL10 and CCL5 ELISA

For evaluation of secreted cytokines, ELISAs for CXCL10 and CCL5 CytoSet (both Invitrogen) were used. The protocols of the manufacturers were followed with a few exceptions. RPMI (Lonza, Basel, Switzerland) with P&S (Invitrogen, Glostrup, Denmark) was used in the standard curve instead of assay buffer, PBS pH 7.2–7.4 (Lonza, Basel, Switzerland) was used as coating buffer A, and TBM X-tra (Kem-En-Tec Diagnostics, Taastrup, Denmark) was used as substrate solution. After TBM X-tra (Kem-En-Tec Diagnostics, Taastrup, Denmark) was added according to protocol, the reaction was stopped after 7–10 min with 0.5 M H_2_SO_4_ (Th. Geyer, Renningen, Germany) instead of the stop solution. The measurements of the absorbance were made on a FluoStar Omega (BMG LABTECH GmbH, Ortenberg, Germany) at 450 nm with 650 nm as reference absorbance, and a 4-parameter curve fit was used in the formation of the standard curve. The lower level of detection was 15.6 pg/mL with the standard curve ranging up to 1000 pg/mL.

### 2.7. RNA Isolation

Trizol (Invitrogen, Glostrup, Denmark) and cell mix harvested from the experiments was thawed, and chloroform (Merck, Darmstadt, Germany) was added. The mixture was centrifuged at 12,000 rpm for 12–15 min at 4°C. Next the RNA/DNA phase was transferred to new tubes and isopropanol (Merck, Darmstadt, Germany) was added 1 : 1. The mixture was incubated for 10 min and then centrifuged at 12,000 rpm for 10–20 min at 4°C. The RNA/DNA precipitated in the bottom of the tube which was then washed with 75% ethanol. The ethanol was removed, and the pellet air dried before it was dissolved in nuclease free water (VWR, Herlev, Denmark).

### 2.8. Measurement of RNA Expression by qPCR

To measure RNA transcription, the total unamplified RNA was DNase I treated (Ambion, Austin, USA) and reverse transcribed using oligo-d(T), dNTP, and M-MLV Reverse Transcriptase (all Invitrogen, Glostrup, Denmark). The cDNA was subjected to qPCR using QuantiFast SYBR Green PCR Kit (Qiagen, Valencia, CA, USA). The following primers were used: ISG56 5′-CCTCCTTGGGTTCGTCTACA-3′ (forward) 5′-GGCTGATATCTGGGTGCCTA-3′ (reverse), Viperin 5′-TGCCACAATGTGGGTGCTTACAC-3′ (forward) 5′-CTCAAGGGGCAGCACAAAGGAT-3′ (reverse), GAPDH 5′-GCAAATTCCATGGCACCGT-3′ (forward) 5′-TCGCCCCACTTGATTTTGG-3′ (reverse). The program used was 95°C in 5 min followed by 45 cycles with 95°C in 10 sec., 62°C in 25 sec., and during the last cycle, a melting curve was made. Calculation of the relative changes in RNA transcription was made using GAPDH as reference gene and the CTR values as control.

## 3. Results

### 3.1. Influence of Differentiation Procedure on Morphological Characteristics of MDMs

**Figure 1:**
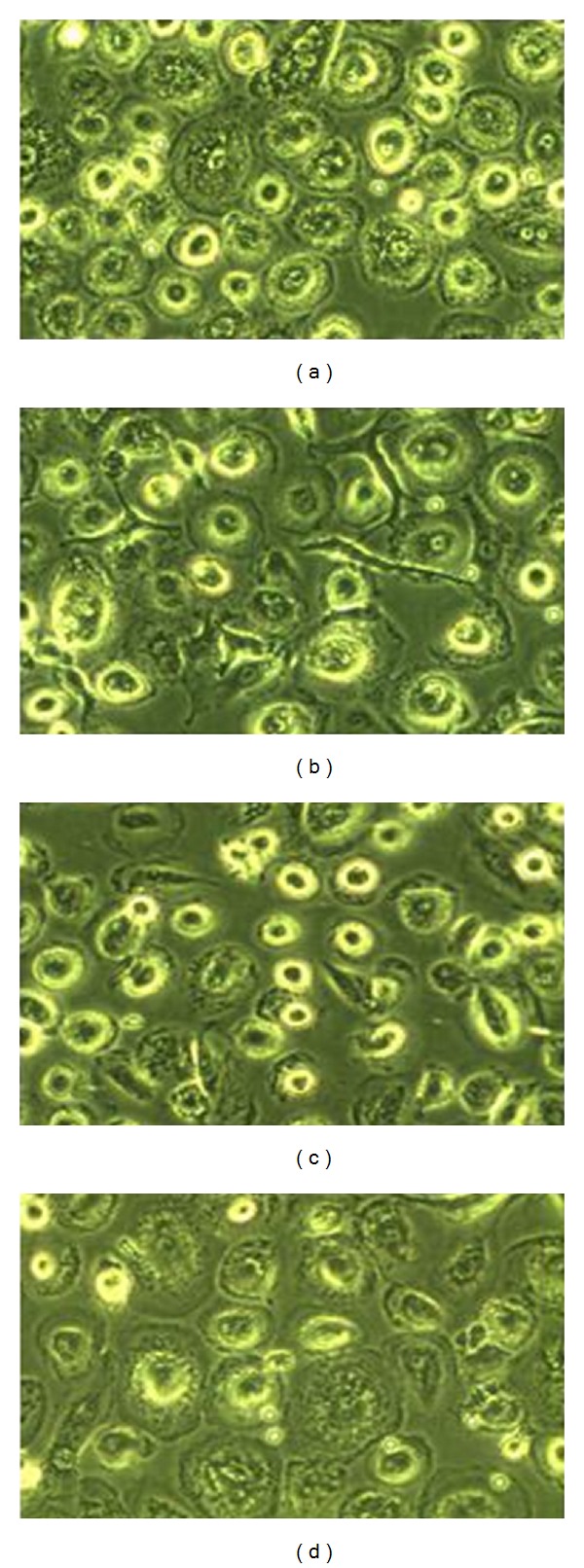
Morphology of MDMs differentiated by four distinct protocols. Representative images of MDMs differentiated for 7 days with (a) RPMI GM-CSF, (b) RPMI M-CSF, (c) Mac-SFM GM-CSF, or (d) Mac-SFM M-CSF.

The MDMs displayed different morphological characteristics dependent on the differentiation conditions (Figure 1). MDMs differentiated with RPMI GM-CSF had a tendency to be large, round, and granulated, whereas MDMs differentiated with RPMI M-CSF appeared smaller and less granulated and with some of the cells also having branched cytoplasm. The MDMs differentiated with Mac-SFM and GM-CSF had a highly branched and small cytoplasm. Finally, the Mac-SFM M-CSF-differentiated MDMs were larger and particularly granulated in the area surrounding the nucleus. Although we observed some donor differences, the morphological characteristics of the MDMs followed the pattern described earlier and were consistently dependent on the differentiation protocol chosen. These observations indicated that the differentiation methods lead to development of phenotypically different MDMs. 

### 3.2. Differential HIV-1 pWT/BaL Interaction and Replication in MDMs

**Figure 2:**
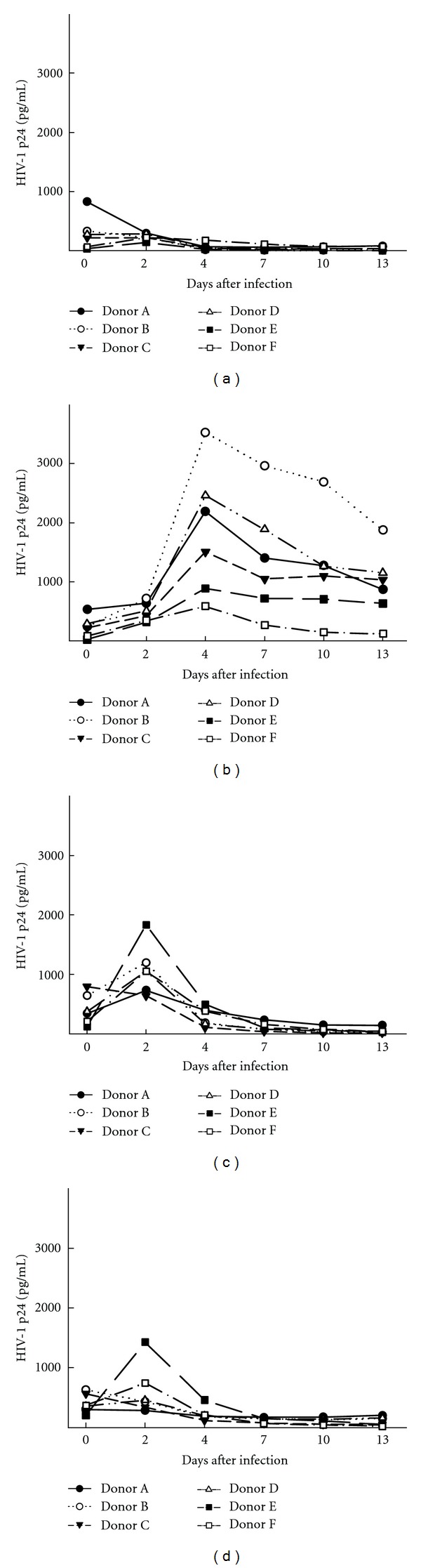
Evaluation of HIV-1 infection and replication in different MDM populations. MDMs were produced during a 7-day period by four different differential protocols and infected with HIV-1 pWT/BaL (5000 × TCID50). Supernatants were harvested at the indicated time points after infection, and p24 levels were evaluated by ELISA. (a) RPMI GM-CSF, (b) RPMI M-CSF, (c) Mac-SFM GM-CSF, and (d) Mac-SFM M-CSF. Data shown are from two different experiments including 6 donors in total.

To investigate the patterns of HIV infection in the different MDM types, a total of 6 donors from two independent experiments were infected with 5,000 × TCID50 of HIV-1 pWT/BaL or pUC19. pUC19, the empty plasmid into which the HIV-1 pWT/BaL was generated, was included as a negative control for possible contamination from the 293 T cells used during virus preparation. The washing controls were produced by incubating MDMs with HIV-1 pWT/BaL for 4 hours, then washing cells three times with PBS, followed by addition of the relevant medium which was then harvested immediately after addition. The washing controls were marked as day 0 in Figure 2. The remaining supernatants were harvested in intervals of either two or three days as indicated in Figure 2, showing the level of HIV-1 secreted from the cells as a function of days of infection. The supernatants harvested from MDMs differentiated with the same medium type were from the same well. The experiments demonstrated major differences in the way the MDMs differentiated with the various media (RPMI versus Mac-SFM) and growth factors (M-CSF versus GM-CSF) responded to the presence of HIV-1 pWT/BaL (Figure 2). The MDMs differentiated with RPMI1640 and M-CSF showed continuing replication of HIV-1 pWT/BaL up till day 4 after infection, after which time the p24 levels slowly decreased (Figure 2(b)). Approximately, the same pattern was found in all the donors, and none of the MDMs produced by using the other differentiation methods showed the same continuing release of virus particles (Figures 2(a), 2(c), and 2(d)). In conclusion, RPMI M-CSF-differentiated MDMs support HIV-1 replication (Figure 2(b)). 

Infection of Mac-SFM GM-CSF and Mac-SFM M-CSF MDMs with HIV-1 pWT/BaL showed an early peak in the released level of viral p24 at day 2 after infection (Figures 2(c) and 2(d)). Importantly, release of virus particles occurred after the completion of the washing procedure and before the harvest on day two in all but one of the donors. These data indicate the existence of some modest time-limited replication or selective uptake and release of virus particles in these types of MDM, which did not result in continuous HIV replication. MDMs differentiated using RPMI containing GM-CSF did not show any p24 production during HIV-1 pWT/BaL infection (Figure 2(a)), suggesting that these cells are resistant to infection by HIV pWT/BaL. 

### 3.3. Influence of Differentiation Procedure on Innate TLR Responses in MDMs

**Figure 3:**
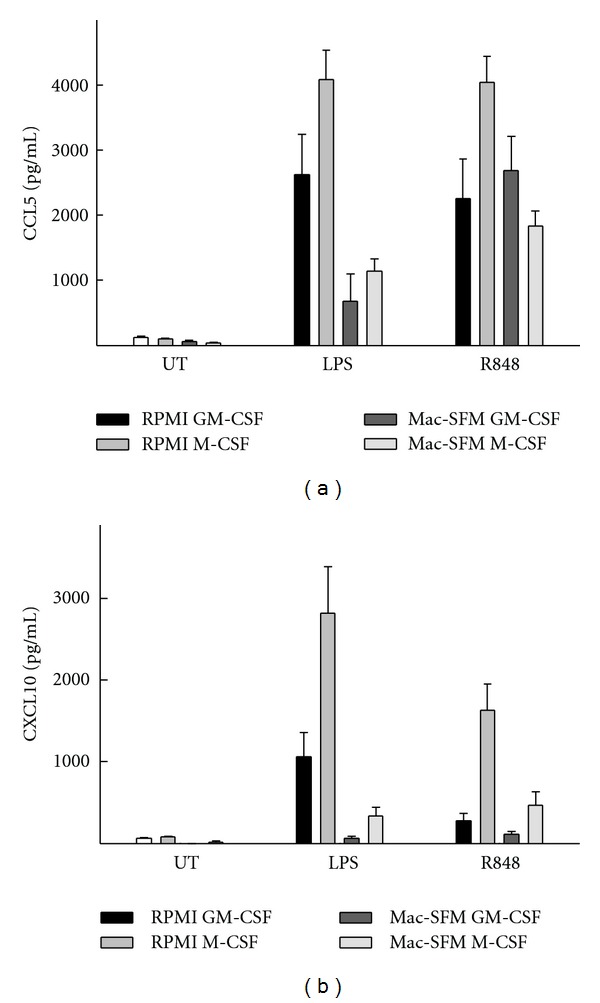
Innate sensing of PAMPs in human MDMs via TLR4 and TLR7/8. MDMs were produced during a 7-day period through stimulation by four differentiation protocols. The different MDM types were stimulated with either LPS (100 ng/ml), R848 (5 *μ*g/mL), or left untreated (UT) for 6 hours before isolation of supernatants. Levels of CCL5 and CXCL10 were measured by ELISA. Data shown are mean values ± SEM from 10 donors from 3 separate experiments.

In order to compare the ability of MDMs to support viral replication to the level of innate immune activation and particularly cellular responsiveness to TLR agonists, we stimulated the four types of MDM with PAMPs (Figure 3). We focused on PAMPs possibly relevant during HIV infection, that is, LPS from translocated bacteria and TLR7/8 agonist mimicking recognition of HIV RNA [24, 26]. The production of the chemokines CXCL10 and CCL5 was measured as markers of innate immune activation [37, 38]. 

To characterize the general immune activation state of the MDMs, we first used LPS to induce an innate immune response through the TLR4 pathway. The strongest induction of CXCL10 and CCL5 by LPS was observed in RPMI M-CSF-differentiated MDMs (Figures 3(a) and 3(b)). When comparing GM-CSF- and M-CSF-differentiated MDMs, we found that M-CSF-differentiated MDMs combined with either RPMI or Mac-SFM secreted higher levels of CXCL10 and CCL5 compared to secretion from GM-CSF-differentiated MDMs.

Next, we stimulated MDMs with the TLR7/8 agonist R848. We found that CCL5 induction after R848 stimulation slightly differed from the pattern following LPS stimulation (Figure 3(a)). We observed a robust and almost equal CCL5 response in all 4 types of MDMs, with a tendency towards a higher response by the RPMI M-CSF MDMs. The pattern of CXCL10 induction after R848 stimulation was similar to what we observed after LPS stimulation, that is with the highest response in RPMI M-CSF-differentiated MDMs (Figure 3(b)). Collectively, the data suggest that RPMI M-CSF-differentiated MDMs are more responsive to TLR stimulation, at least through TLR4 and TLR7/8. 

### 3.4. Influence of Differentiation Procedure on Innate Response to Cytoplasmic DNA in MDMs

**Figure 4:**
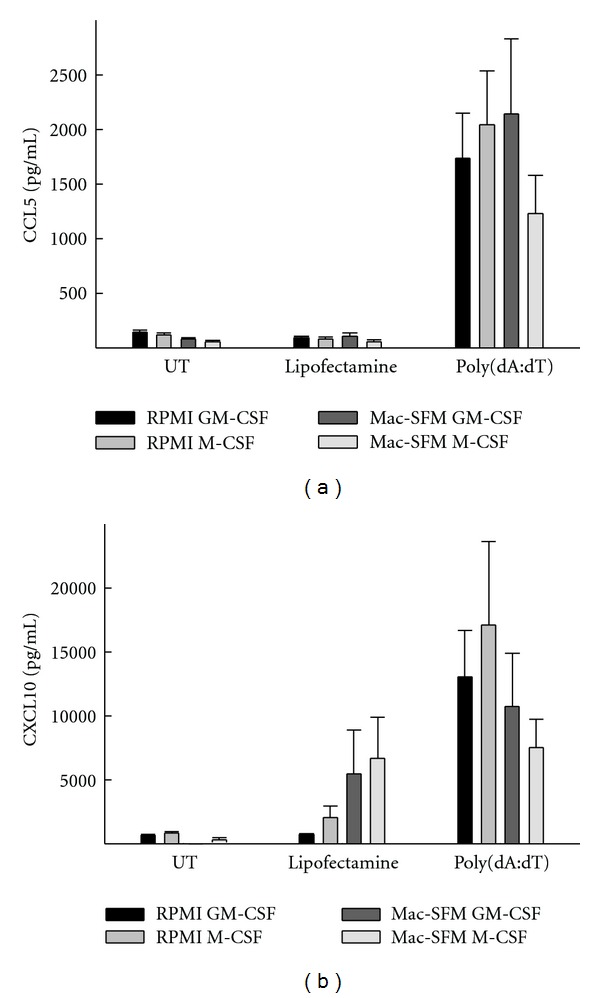
Innate sensing of DNA in human MDMs via intracellular DNA sensors. MDMs were produced during a 7-day period through stimulation by four differentiation protocols. The MDMs were transfected with poly(dA : dT) (1.7 *μ*g/mL) using lipofectamine2000, treated with lipofectamine alone, or left untreated (UT) for 6 hours before harvest of supernatants. Levels of CCL5 and CXCL10 were measured by ELISA. Data shown are means ± SEM from 6 donors from 2 separate experiments.

DNA is formed during reverse transcription of the viral genome and has also been suggested to be sensed by innate immune cells during HIV infection [23, 25]. To investigate whether MDMs recognize DNA, we transfected MDMs with synthetic DNA in the form of poly(dA : dT). As shown in Figures 4(a) and 4(b), induction of CCL5 and CXCL10 by poly(dA : dT) was somewhat similar in all 4 MDM types irrespective of the differentiation procedure, although the response was lower in Mac-SFM/M-CSF MDMs and tended to be higher in RPMI/M-CSF MDMs. Lipofectamine2000 was used for transfection of DNA into the MDMs, and as shown in Figure 4(b), empty liposomes did induce CXCL10 production, especially in MDMs differentiated with Mac-SFM, hence indicating activation of the recently identified innate pathway triggered by membrane fusion [39]. In summary, the data show that all types of MDMs sense DNA resulting in innate immune activation and also indicate that Mac-SFM-differentiated MDMs respond to membrane fusion with expression of CXCL10.

### 3.5. Induction of ISGs by HIV-1 MDMs Differentiated with RPMI and M-CSF

**Figure 5:**
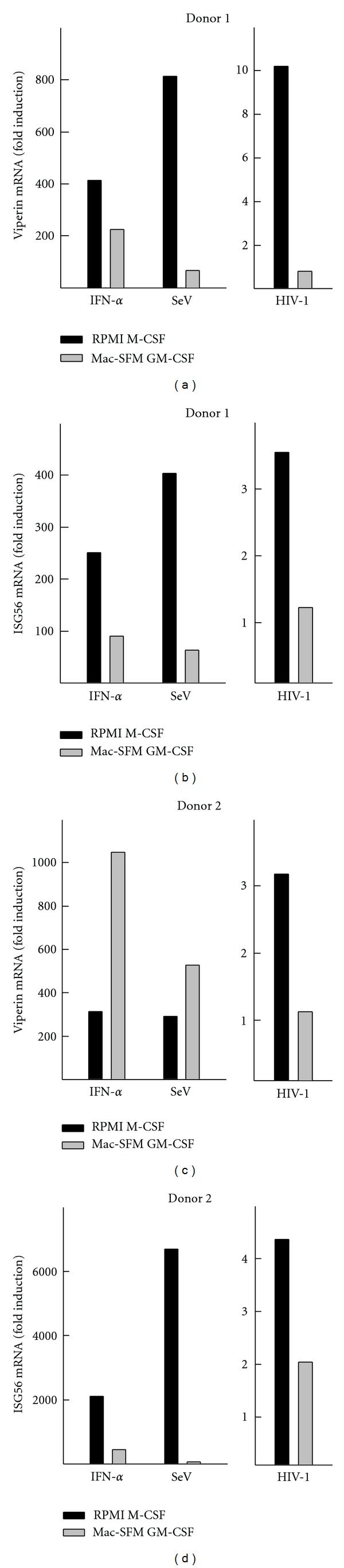
Induction of ISGs by HIV-1 in human MDMs. MDMs were produced during a 7-day period through two differentiation protocols (RPMI M-CSF and Mac-SFM GM-CSF). MDMs were treated with IFN-*α* (100 U/mL), Sendai virus (SeV) (0.5 MOI), HIV-1 pWT/BaL (5000 × TCID50), or left untreated for 6 hours before total RNA was harvested and analysed for viperin (a) and (c) and ISG56 (b) and (d) expression by PCR. Data are shown from two donors. Data are normalized to GAPDH and shown as fold induction relative to untreated.

After investigating the general innate immune activating capability of the four types of MDMs, we wished to investigate any possible immune activation after addition of HIV-1 pWT/BaL. For this purpose, we used RPMI M-CSF and Mac-SFM GM-CSF MDMs.  Figure 5 shows fold induction (relative to mock) of normalized levels of the two selected ISGs viperin (Figures 5(a) and 5(c)) and ISG56 (Figures 5(b) and 5(d)) with known antiviral functions [40, 41] in RNA harvested 6 h after stimulation with IFN-*α* or infection with Sendai virus or HIV-1 pWT/BaL. We observed that Sendai virus, which stimulates ISG expression via RIG-I [42], induced much higher responses in the RPMI M-CSF MDMs as compared to the Mac-SFM GM-CSF MDMs (Figure 5). With respect to HIV-1 infection, we found reproducible induction of viperin and ISG56 in RPMI M-CSF-differentiated MDMs from 2 out of 4 donors after HIV infection. In contrast, the Mac-SFM GM-CSF MDMs displayed no stimulation of viperin or ISG56 transcription in HIV-1 pWT/BaL infected cells. These results indicate that in some donors, HIV-1 induces elevated transcription of viperin and ISG56 and that this depends on the specific type of MDMs, since this was only observed in RPMI M-CSF differentiated MDMs. 

## 4. Discussion

Macrophages are central players in the orchestration of efficient innate immune responses, including virus infections [43]. During HIV infection, macrophages serve as viral reservoir and also contribute to chronic immune activation by inducing proinflammatory mediators, which is central in the development of progressive immunodeficiency [21]. Therefore, knowledge of the interaction between HIV and macrophages is of great importance for understanding how HIV-1 suppresses innate immune recognition to allow establishment of viral reservoirs, as well as the role of macrophages in generating a state of chronic immune activation. Here, we describe MDMs generated by different differentiation protocols and evaluate how this influences macrophage properties, including HIV-1 infection kinetics and p24 production, innate immune responses to well-defined PAMPs, and finally HIV-induced ISG expression.

A number of different protocols have classically been used to differentiate MDMs from primary PBMCs for in vitro experiments. These differences may include variations in the medium, choice of growth factors, serum type, and the duration of the differentiation period [10, 19–21]. Differential generation of these types of MDMs may influence the functional properties of the cells including permissiveness to infections and response to PRR agonists [3, 4, 13]. One key finding of the study was the observation of elevated and relatively persistent HIV-1 pWT/BaL replication in MDMs differentiated with RPMI1640 and M-CSF. This suggests that this type of MDM could be useful as a model system for HIV-1 replication in primary human macrophages. Such a model for HIV replication is a prerequisite for studying the interactions between HIV-1 and the innate immune system in macrophages during HIV-1 replication. Currently, some of the areas of uncertainty and focus of interest in this field are sensing of HIV PAMPs by PRRs. Data published so far suggest that important cell type differences exist, which may be partly explained by differential expression of PRRs among different innate immune cells. Whereas TLR7 sensing of HIV ssRNA has been demonstrated to take place in plasmacytoid DCs [24, 44, 45], a role for cytosolic RLRs for recognition of HIV genomic RNA in PBMCs has been suggested by work from our laboratory and others [2, 10]. Moreover, cytosolic DNA sensors may also be operative in certain cell types, including macrophages, during HIV infection [23]. 

A general consideration is to identify the molecular differences between the MDM types with different permissiveness for HIV-1 infection (e.g., RPMI M-CSF-differentiated versus RPMI GM-CSF-differentiated). Our data may suggest that HIV-1 pWT/BaL entry is via CD4-coreceptor uptake in M-CSF RPMI-differentiated MDMs resulting in productive viral infection, whereas viral entry may be via receptor-independent endocytosis in RPMI GM-CSF differentiated MDMs with subsequent absence or only very limited viral replication. This issue is likely very complex, but may include differential expression of HIV-1 receptor molecules and PRRs. To address the latter question, we investigated the responsiveness of the different MDMs to PAMPs targeting PRRs of relevance for HIV-1 infection. We found that RPMI M-CSF-differentiated MDMs stimulated gene expression more potently through TLR4 and TLR7/8 than RPMI GM-CSF-differentiated MDMs, and we also observed that the RPMI M-CSF-differentiated MDMs exhibited more potent stimulation of the RLR pathway than Mac-SFM GM-CSF-differentiated MDMs. By contrast, the MDM types exhibited comparable responsiveness to stimulation with intracellular DNA. As an incidental finding, we observed that Mac-SFM MDMs tended to respond more potently to membrane fusion-inducing liposomes, which has recently been described as an novel cellular mechanism for activating innate immune responses [39, 46]. The finding that the RPMI M-CSF-differentiated MDMs in our experimental setup produced the highest amounts of proinflammatory chemokines indicate that this differentiation protocol results in MDMs with a high potential to produce proinflammatory mediators after PAMP stimulation than GM-CSF-differentiated MDMs. Importantly, our results indicates that there may be a direct correlation between the marked responsiveness to TLR stimulation and the ability of HIV-1 pWT/BaL to establish productive infection with relatively robust p24 levels. However, in this study, we did not combine HIV infection and PAMP stimulation, which may have provided further insight into the direct effect of PRR stimulation on HIV replication in macrophages.

It has been previously reported that shortly after infection with HIV-1, certain ISGs are upregulated [41]. Therefore, we investigated the differences in viperin and ISG56 expression in RPMI M-CSF and Mac-SFM GM-CSF-differentiated MDMs after infection with HIV-1 pWT/BaL. Interestingly, we were able to reproducibly detect induction of viperin mRNA expression in RPMI M-CSF-differentiated MDMs, in 2 of 4 donors (Figure 5). This finding is in line with previous suggestions that viperin may contribute to persistent noncytopathic HIV infection of macrophages [41]. In our study, viperin was not induced in Mac-SFM-differentiated MDMs in neither of the donors. This indicates that viperin induction depends either on productive HIV infection or alternatively may be attributed to some features possessed by the RPMI M-CSF- but not the Mac-SFM-differentiated MDMs. In addition, we also measured levels of CXCL10 and CCL5, at various time points following HIV-1 pWT/BaL infection, and consistently were not able to measure induction of these cytokines (data not shown). These absent or very minimal cellular responses are thus in agreement with previous studies by others demonstrating that HIV-1 is able to prevent expression of proinflammatory cytokines and antiviral type I IFN responses in macrophages [7].

If the assumption that RPMI M-CSF-differentiated MDMs can be used as a good model system is correct, further studies should be focused on searching for relevant read outs (i.e., inflammatory cytokines, chemokines, and IFN) of an innate immune response. This would allow much more modulation of the system in terms of studying pathogen sensing, signaling pathways and so forth. The advantages of using RPMI M-CSF differentiated MDMs as a model for in vivo macrophages also supported by previous reports of high levels of circulating M-CSF in normal blood performing a role as a homeostatic growth factor [22]. It is therefore possible that the differentiation of macrophages in vivo is drifting toward a phenotype similar to the phenotype generated in vitro in the present study using RPMI M-CSF. This differentiation method may mimic the tissue macrophage present in humans during HIV infection in vivo and thus provide an appropriate picture of the physiological situation. By contrast, in situations where it may be desirable to study very early steps of HIV infection without establishment of productive infection and continuous viral production, for example, when studying upstream sensing of HIV PAMPs, using model systems using Mac-SFM with either M-CSF or GM-CSF may be advantageous. Indeed, it has been previously proposed that M-CSF-differentiated macrophages may be used to study tissue macrophage functions, whereas in contrast, GM-CSF-differentiated macrophages are more DC-like [14–16], which correlates with of observations in the present work of a very limited ability of HIV to establish infection in this cell type. It should also be noted that we, in line with others, observed major donor differences in the susceptibility to HIV-1 pWT/BaL infection and in PRR responsiveness. This high degree of variability in the ability of macrophages to support HIV replication has been suggested to be partly due to genetic variations that may contribute to susceptibility to infection [47].

In line with previous studies, our study demonstrated that HIV does not evoke a strong innate immune response upon infection of primary macrophages, although we were able to detect virus-induced expression of the ISG viperin in some donors. Thus, HIV-infected macrophages do not produce significant amounts of type I IFN, ISGs, or proinflammatory cytokines but instead evade recognition by the immune system allowing them to serve as a viral reservoir. Despite this, a subset of ISGs is now known to restrict HIV replication, hence suggesting that some innate signaling is activated and potentially targeted by the virus in macrophages [48, 49]. More in-depth understanding of the molecular mechanisms behind the HIV-macrophage interactions may provide insight into the pathogenesis of immune dysfunction and development of immunodeficiency during HIV infection. 

## 5. Conclusions

In conclusion, our study demonstrated major differences between MDMs differentiated through different protocols in their ability to support HIV replication and evoke innate immune activation in response to PAMPs and HIV-1 infection. Overall, macrophages do not evoke a strong innate immune response. Paradoxically, the data show that the MDMs with the strongest innate immune response were also the most permissive for HIV-1 replication. Identification of cellular factors affecting HIV entry and replication in macrophages will provide insight into the interactions between HIV and macrophages and the role played by this important innate cell type in the pathogenesis of HIV infection. 

## References

[1] Herbeuval JP, Shearer GM (2007). HIV-1 immunopathogenesis: how good interferon turns bad. *Clinical Immunology*.

[2] Berg RK, Melchjorsen J, Rintahaka J (2012). Genomic HIV RNA induces innate immune responses through RIG-I-dependent sensing of secondary-structured RNA. *PloS One*.

[3] Cobos-Jimenez V, Booiman T, Hamann J (2011). Macrophages and HIV-1. *Current Opinion in HIV & AIDS*.

[4] Borel S, Espert L, Biard-Piechaczyk M (2012). Macroautophagy regulation during HIV-1 infection of CD4+ T cells and macrophages. *Frontiers in Immunology*.

[5] Mogensen SC (1979). Role of macrophages in natural resistance to virus infections. *Microbiological Reviews*.

[6] Wang X, Chao W, Saini M, Potash MJ (2011). A common path to innate immunity to HIV-1 induced by Toll-like receptor ligands in primary human macrophages. *PloS One*.

[7] Tsang J, Chain BM, Miller RF (2009). HIV-1 infection of macrophages is dependent on evasion of innate immune cellular activation. *AIDS*.

[8] Malim MH, Bieniasz PD (2012). HIV restriction factors and mechanisms of evasion. *Cold Spring Harbor Perspectives in Medicine*.

[9] Manel N, Littman DR (2011). Hiding in plain sight: how HIV evades innate immune responses. *Cell*.

[10] Solis M, Nakhaei P, Jalalirad M (2011). RIG-I-mediated antiviral signaling is inhibited in HIV-1 infection by a protease-mediated sequestration of RIG-I. *Journal of Virology*.

[11] Doehle BP, Chang K, Rustagi A (2012). Vpu mediates depletion of interferon regulatory factor 3 during HIV infection by a lysosome-dependent mechanism. *Journal of Virology*.

[12] Rich EA, Chen ISY, Zack JA, Leonard ML, O’Brien WA (1992). Increased susceptibility of differentiated mononuclear phagocytes to productive infection with human immunodeficiency virus-1 (HIV-1). *Journal of Clinical Investigation*.

[13] Lacey DC, Achuthan A, Fleetwood AJ (2012). Defining GM-CSF- and macrophage-CSF-dependent macrophage responses by in vitro models. *The Journal of Immunology*.

[14] Akagawa KS (2002). Functional heterogeneity of colony-stimulating factor-induced human monocyte-derived macrophages. *International Journal of Hematology*.

[15] Verreck FAW, De Boer T, Langenberg DML (2004). Human IL-23-producing type 1 macrophages promote but IL-10-producing type 2 macrophages subvert immunity to (myco)bacteria. *Proceedings of the National Academy of Sciences of the United States of America*.

[16] Fleetwood AJ, Lawrence T, Hamilton JA, Cook AD (2007). Granulocyte-macrophage colony-stimulating factor (CSF) and macrophage CSF-dependent macrophage phenotypes display differences in cytokine profiles and transcription factor activities: Implications for CSF blockade in inflammation. *Journal of Immunology*.

[17] Peters PJ, Bhattacharya J, Hibbitts S (2004). Biological analysis of human immunodeficiency virus type 1 R5 envelopes amplified from brain and lymph node tissues of AIDS patients with neuropathology reveals two distinct tropism phenotypes and identifies envelopes in the brain that confer an enhanced tropism and fusigenicity for macrophages. *Journal of Virology*.

[18] Nguyen DG, Hildreth JEK (2003). Involvement of macrophage mannose receptor in the binding and transmission of HIV by macrophage. *European Journal of Immunology*.

[19] Luban J (2012). Innate immune sensing of HIV-1 by dendritic cells. *Cell Host and Microbe*.

[20] Gobeil LA, Lodge R, Tremblay MJ (2012). Differential HIV-1 endocytosis and susceptibility to virus infection in human macrophages correlate with cell activation status. *Journal of Virology*.

[21] Mogensen TH, Melchjorsen J, Larsen CS, Paludan SR (2010). Innate immune recognition and activation during HIV infection. *Retrovirology*.

[22] Brown JN, Kohler JJ, Coberley CR, Sleasman JW, Goodenow MM (2008). HIV-1 activates macrophages independent of toll-like receptors. *PLoS ONE*.

[23] Yan N, Regalado-Magdos AD, Stiggelbout B, Lee-Kirsch MA, Lieberman J (2010). The cytosolic exonuclease TREX1 inhibits the innate immune response to human immunodeficiency virus type 1. *Nature Immunology*.

[24] Meier A, Alter G, Frahm N (2007). MyD88-dependent immune activation mediated by human immunodeficiency virus type 1-encoded toll-like receptor ligands. *Journal of Virology*.

[25] Doitsh G, Cavrois M, Lassen KG (2010). Abortive HIV infection mediates CD4 T cell depletion and inflammation in human lymphoid tissue. *Cell*.

[26] Brenchley JM (2006). Microbial translocation is a cause of systemic immune activation in chronic HIV infection. *Nature Medicine*.

[27] Pine SO, McElrath MJ, Bochud PY (2009). Polymorphisms in toll-like receptor 4 and toll-like receptor 9 influence viral load in a seroincident cohort of HIV-1-infected individuals. *AIDS*.

[28] Hernandez JC, Arteaga J, Paul S (2011). Up-regulation of TLR2 and TLR4 in dendritic cells in response to HIV type 1 and coinfection with opportunistic pathogens. *AIDS Research and Human Retroviruses*.

[29] Gringhuis SI, Van Der Vlist M, Van Den Berg LM, Den Dunnen J, Litjens M, Geijtenbeek TBH (2010). HIV-1 exploits innate signaling by TLR8 and DC-SIGN for productive infection of dendritic cells. *Nature Immunology*.

[30] Báfica A, Scanga CA, Schito M, Chaussabel D, Sher A (2004). Influence of coinfecting pathogens on HIV expression: evidence for a role of Toll-like receptors. *Journal of Immunology*.

[31] Kornbluth RS, Oh PS, Munis JR, Cleveland PH, Richman DD (1989). Interactions and bacterial lipopolysaccharide protect macrophages from productive infection by human immunodeficiency virus in vitro. *Journal of Experimental Medicine*.

[32] Mancino G, Placido R, Bach S (1997). Infection of human monocytes with Mycobacterium tuberculosis enhances human immunodeficiency virus type 1 replication and transmission to T cells. *Journal of Infectious Diseases*.

[33] Ahmed N, Hayashi T, Hasegawa A (2010). Suppression of human immunodeficiency virus type 1 replication in macrophages by commensal bacteria preferentially stimulating Toll-like receptor 4. *Journal of General Virology*.

[34] Pirhonen J, Sareneva T, Kurimoto M, Julkunen I, Matikainen S (1999). Virus infection activates IL-1*β* and IL-18 production in human macrophages by a caspase-1-dependent pathway. *Journal of Immunology*.

[35] Österlund P, Veckman V, Sirén J (2005). Gene expression and antiviral activity of alpha/beta interferons and interleukin-29 in virus-infected human myeloid dendritic cells. *Journal of Virology*.

[36] Kirkegaard T, Wheatley A, Melchjorsen J (2011). Induction of humoral and cellular immune responses against the HIV-1 envelope protein using gamma-retroviral virus-like particles. *Virology Journal*.

[37] Reimer T, Brcic M, Schweizer M, Jungi TW (2008). poly(I:C) and LPS induce distinct IRF3 and NF-*κ*B signaling during type-I IFN and TNF responses in human macrophages. *Journal of Leukocyte Biology*.

[38] Mantovani A, Sica A, Sozzani S, Allavena P, Vecchi A, Locati M (2004). The chemokine system in diverse forms of macrophage activation and polarization. *Trends in Immunology*.

[39] Holm CK, Jensen SB, Jakobsen MR (2012). Virus-cell fusion as a trigger of innate immunity dependent on the adaptor STING. *Nature Immunology*.

[40] Liu MQ, Zhou D-J, Wang X (2012). IFN-lambda3 inhibits HIV infection of macrophages through the JAK-STAT pathway. *PLoS One*.

[41] Nasr N, Susan M, Stuart GT (2012). HIV-1 infection of human macrophages directly induces viperin which inhibits viral production. *Blood*.

[42] Kato H, Takeuchi O, Sato S (2006). Differential roles of MDA5 and RIG-I helicases in the recognition of RNA viruses. *Nature*.

[43] Kumagai Y, Takeuchi O, Kato H (2007). Alveolar macrophages are the primary interferon-*α* producer in pulmonary infection with RNA viruses. *Immunity*.

[44] Lepelley A, Louis S, Sourisseau M (2011). Innate sensing of HIV-infected cells. *PLoS Pathog*.

[45] Beignon AS, McKenna K, Skoberne M (2005). Endocytosis of HIV-1 activates plasmacytoid dendritic cells via Toll-like receptor-viral RNA interactions. *Journal of Clinical Investigation*.

[46] Søby S, Laursen RR, Østergaard L (2012). HSV-1-induced chemokine expression via IFI16-dependent and IFI16-independent pathways in human monocyte-derived macrophages. *Herpesviridae*.

[47] Bol SM, van Remmerden Y, Sietzema JG, Kootstra NA, Schuitemaker H, van ’t Wout AB (2009). Donor variation in in vitro HIV-1 susceptibility of monocyte-derived macrophages. *Virology*.

[48] Jakobsen MR, Mogensen TH, Paludan SR Caught in translation: innate restriction of HIV mRNA translation by a schlafen family protein.

[49] Li M, Kao E, Gao X (2012). Codon-usage-based inhibition of HIV protein synthesis by human schlafen 11. *Nature*.

